# Residual Renal Risk in Diabetic Nephropathy Despite Contemporary Therapies

**DOI:** 10.3390/jcm15030921

**Published:** 2026-01-23

**Authors:** Reinhart Speeckaert, Charlotte Delrue, Marijn M. Speeckaert

**Affiliations:** 1 Department of Dermatology, Ghent University Hospital, 9000 Ghent, Belgium; reinhart.speeckaert@uzgent.be; 2 Department of Nephrology, Ghent University Hospital, 9000 Ghent, Belgium; 3 Research Foundation-Flanders (FWO), 1000 Brussels, Belgium

**Keywords:** diabetic kidney disease, residual renal risk, chronic kidney disease progression, renoprotective therapies

## Abstract

**Background/Objectives:** Diabetic kidney disease (DKD) is the most prevalent form of chronic kidney disease and kidney failure globally. In the last decade, RAAS inhibitors, SGLT2 inhibitors, GLP-1-receptor agonists, and non-steroidal mineralocorticoid receptor antagonists have made significant advancements. However, despite the use of these drugs, patients with DKD often show a residual renal risk. In this narrative review, we synthesize current evidence on residual renal risk in DKD, focusing on underlying mechanisms, high-risk clinical phenotypes, and therapeutic gaps. **Methods:** A narrative review of current literature available in clinical trials, post hoc analysis studies, observational studies, and research into mechanisms of action or processes related to DKD was performed. **Results:** The central element of residual renal risk in DKD is persistent inflammation, fibrosis, tubulointerstitial injury/injury to both renal and vascular systems, and metabolic memory. These represent the four types of renal-related problems that have been identified. Non-albuminuric DKD, rapid progressors, late presenters with multiple comorbidities, tend to exhibit an extreme burden associated with some of these entities. **Conclusions:** The remaining risk factors that cannot be adequately addressed by current medical treatments represent major opportunities for improved clinical management, i.e., those at high-risk of kidney failure. Future research initiatives are required to further refine and improve therapeutic approaches for early intervention in patients with DKD. In addition, development of specific treatments targeting inflammation, fibrosis and metabolism in the kidney will lead to better therapeutic results and decreased risk of progressive loss of kidney function.

## 1. Introduction

Diabetic kidney disease (DKD) is the most common cause of chronic kidney disease (CKD) and kidney failure worldwide, representing about 40–50% of patients beginning kidney replacement therapy in many developed countries [1,2]. Despite significant advances in treating diabetes and its complications, DKD still poses a considerable clinical and economic burden, caused by the gradual decline in kidney function, high rates of cardiovascular complications, and excess mortality [3,4]. The projection estimates that the number of individuals with DKD will increase in tandem with the growing incidence of type 2 diabetes, the aging population, and the longer life span of patients with an extended history of type 2 diabetes [5].

The way DKD is treated has undergone drastic changes over the last ten years regarding the best approach to managing a person with DKD. For many years, blocking the renin–angiotensin–aldosterone system (RAAS) with either an angiotensin-converting enzyme inhibitor (ACE-I) or an angiotensin II receptor blocker (ARB) was regarded as the gold standard in providing renoprotection for DKD patients due to their proven ability to reduce albuminuria and slow the rate of decline in estimated glomerular filtration rate (eGFR) [6]. More recently, using sodium-glucose cotransporter 2 (SGLT2) inhibitors has become an important part of DKD treatment and has a strong and consistent effect on decreasing one’s risk of developing sustained declines in eGFR, kidney failure, and death due to kidney disease, across a wide range of baseline kidney function and albumin levels [7,8]. Glucagon-like peptide-1 receptor agonists (GLP-1 RAs) have also demonstrated beneficial effects on the kidney, including reducing albuminuria and cardiovascular events [9]. The non-steroidal mineralocorticoid receptor antagonist (nsMRA) finerenone has added incremental kidney and cardiovascular protection beyond that provided by blocking the RAAS for DKD patients [10,11].

Despite these advances, DKD remains progressive in a substantial proportion of patients. Even in randomized controlled trials that include RAAS inhibition, SGLT2 inhibitors, optimized glycemic control, and aggressive blood pressure management, a considerable residual risk of kidney disease progression persists [7,8]. This observation has led to growing recognition of residual renal risk, defined as the ongoing risk of adverse renal outcomes, including sustained eGFR decline, progression to kidney failure, or renal death, despite guideline-directed, evidence-based therapies [12]. This definition serves as the conceptual foundation for the present review and is not reiterated in subsequent sections. Instead, later sections build on this framework to examine evidence, mechanisms, phenotypes, and clinical implications.

Persistent residual renal risk has important clinical implications, highlighting the limitations of current treatment strategies that predominantly target glomerular hemodynamics and glycemic control while incompletely addressing inflammation, tubulointerstitial injury, microvascular dysfunction, and fibrosis. Residual renal risk also reflects the marked heterogeneity of DKD, whereby patients with similar albuminuria and eGFR may experience substantially different trajectories of kidney function decline [13,14,15,16,17]. In addition to identifying the need for broader monitoring of patients with progressive manifestations of nephropathy, persistent renal risk has many clinical implications for monitoring patients, using clinical data when providing patient advice, and designing future nephrology clinical trials. Specifically, a decrease in albuminuria and stabilization of eGFR over a short period do not guarantee long-term preservation of kidney structure and function [18].

Importantly, residual renal risk is not limited to patients with advanced DKD. Post hoc analyses of major outcome trials and large observational cohorts indicate that progressive loss of kidney function can occur even in early-stage disease and in patients with well-controlled glycemia and blood pressure [19,20]. This pattern is particularly evident among individuals with non-albuminuric DKD phenotypes, a history of acute kidney injury (AKI), long diabetes duration, or delayed initiation of renoprotective therapies [21,22]. Moreover, the concept of “metabolic memory,” whereby prior hyperglycemia induces long-lasting epigenetic and structural changes within the kidney, further contributes to the persistence of renal risk despite subsequent therapeutic optimization [23,24].

Given this context, a detailed evaluation of residual renal risk in DKD is warranted to clarify why disease progression persists despite contemporary therapy and to identify patients most likely to benefit from intensified or novel interventions. In addition, it would assist in identifying the next set of therapeutic approaches to pursue.

Importantly, the present review does not aim to provide a comprehensive overview of individual renoprotective drug classes, as these have been extensively addressed in recent international guidelines and consensus statements. Instead, we focus on the concept of residual renal risk as a distinct and persistent clinical entity, synthesizing mechanistic, clinical, and trial-based evidence to explain why kidney disease progression continues despite guideline-directed, multidrug therapy. By integrating pathophysiological mechanisms (persistent inflammation, fibrosis, tubulointerstitial and microvascular injury, and metabolic memory) with high-risk clinical phenotypes and contemporary outcome data, this review proposes a unifying conceptual framework for residual renal risk in DKD. This framework is intended to complement existing literature by shifting the focus from treatment efficacy alone to the biological limits of current therapies and the heterogeneity of disease progression.

The objective of this article is to review the existing literature on residual renal risk in DKD during the era of modern renoprotective therapy and to integrate findings from randomized clinical trials, real-world studies, and translational research to identify the clinical implications of residual renal risk and to suggest methods to further reduce the incidence of kidney disease among diabetic patients.

Throughout this review, conclusions drawn from randomized clinical trials (RCTs) and meta-analyses are distinguished from associations derived from observational studies and mechanistic hypotheses, which are interpreted as hypothesis-generating rather than definitive evidence.

## 2. Methodology

A narrative review was conducted on the existing literature regarding residual kidney risk in individuals with DKD. A literature search was performed in both PubMed/MEDLINE and Embase for articles discussing residual kidney risk, the progression of CKD, renoprotective medications, their mechanism(s) of action (e.g., inflammation, fibrosis, tubulointerstitial injury), and microvascular dysfunction/metabolic memory. The literature search concentrated on published data obtained from RCTs and post hoc analyses of large kidney trial outcome studies, observational cohort studies, and high-quality translational or mechanism-oriented studies that demonstrated that CKD will continue to progress post-DKD therapies. Additionally, published data from current international guidelines, consensus statements, and landmark review articles were utilized to frame and develop this review and to identify gaps in addressing residual renal risk. The literature review primarily consisted of publications released since 2000. However, since most of the current renal protective agents (SGLT2 inhibitors, GLP-1 RAs, and nsMRAs) were developed and introduced during this time period, we chose to use literature published within 10–15 years as a basis for the analysis. Since this is a narrative review, we did not have formal inclusion and exclusion criteria, as well as methods to quantitatively evaluate the likelihood of bias or methodological variances. However, we considered both when synthesizing the data.

## 3. Contemporary Therapies and Their Limitations

### 3.1. Renin–Angiotensin–Aldosterone System (RAAS) Blockade

Long-term renal protection has been a primary goal in treating diabetic nephropathy with RAAS blockade, particularly with ACE-I and ARBs. The ability of both ACE-I and ARBs to slow DKD progression has been established. Specifically, both types of drugs lower intraglomerular pressure and reduce proteinuria, thereby lessening tubulointerstitial damage. The first detailed published results showing NSSH were reported in randomized controlled trials in the 1990s and 2000s. At that time, these trials demonstrated the efficacy of RAAS blockade in delaying the onset of kidney failure and reducing the risk of serum creatinine doubling among patients with albuminuric DKD [6,25,26].

Renoprotection with RAAS blockers is mediated by hemodynamic and non-hemodynamic (immunomodulatory) effects. In the earliest stages of diabetes-related problems, ACE-I and ARBs preferentially dilate the kidneys’ glomerular efferent arterioles. This anti-hyperfiltration effect helps prevent the onset of diabetic kidney injury at the earliest stages of its development. Additionally, by blocking the effects of hormones from the RAAS, these drugs have been shown to inhibit profibrotic and pro-inflammatory signaling involved in transforming growth factor beta (TGF-β) activation, oxidation, and extracellular matrix deposition in the renal tubulo-interstitial regions, thereby protecting these structures from damage [27,28]. It is well established that the degree of albuminuria reduction achieved with RAAS inhibitors is an indicator of improved long-term renal health outcomes [29].

Despite these benefits, several limitations of RAAS blockade have become increasingly evident. ACE inhibitors and ARBs slow the progression of CKD but rarely halt it altogether. Long-term follow-up studies and real-world data have shown that despite RAAS blockade, many patients, especially those with advanced CKD, prolonged duration of diabetes, or high levels of inflammation [30,31]. The risk of further renal injury associated with RAAS inhibition is modest (i.e., a significant amount of renal risk remains even in highly compliant patients).

The effectiveness of RAAS inhibition appears limited to those with DKD who have an albuminuric phenotype. For patients with non-albuminuric DKD, a phenotype that has gained recognition of up to 30–40% of total DKD cases, the renal protective benefits of RAAS inhibitor therapy have been less clear, with studies demonstrating that most of these patients do not experience significant benefits from RAAS inhibitors [17,32]. This presents a challenge to RAAS inhibition as it does not effectively target or treat other causes of acute damage to kidney tissue, such as tubular dysfunction, microvascular rarefaction, and ischemia.

Third, treatment intensification through dual RAAS blockade has proven unsuccessful and potentially harmful. ACE-I combined with ARBs or direct renin inhibitors were initially intended to maximize their antiproteinuric effects. However, trials demonstrated increased incidence of hyperkalemia, hypotension, and acute renal injury while not providing any additional renal benefit [33,34,35]. Therefore, documentation of these data has confirmed that mono-therapy blockade of the RAAS is the standard of care and that there is a very limited therapeutic window within this system. Importantly, attempts to intensify RAAS blockade through dual inhibition or direct renin inhibition not only failed to confer additional renal benefit but were associated with higher rates of adverse events, underscoring the biological and therapeutic ceiling of this pathway.

RAAS blockade is further limited by tolerability and applicability in advanced CKD, where hyperkalemia, early eGFR decline, and hypotension frequently restrict dose optimization or lead to discontinuation [14,36]. As a result, real-world studies consistently show that RAAS inhibitors are often prescribed at submaximal doses or discontinued altogether, further contributing to residual renal risk [37].

Importantly, RAAS blockade does not fully suppress intrarenal RAAS activity. Experimental and clinical studies have demonstrated that alternative pathways of angiotensin II generation, as well as aldosterone escape, may sustain local profibrotic and proinflammatory signaling despite systemic RAAS inhibition [38,39]. This incomplete blockade may partially explain the persistence of kidney injury and progression despite apparently optimal therapy.

Finally, RAAS inhibitors were developed and validated in an era preceding the widespread recognition of DKD heterogeneity and the availability of newer disease-modifying agents. RAAS inhibitors are the basis of therapy, though they mainly focus on improving glomerular blood flow and reducing albuminuria. However, they do not adequately address several other important ways to stop or slow down DKD, including metabolic memory, mitochondrial problems, immune system activation, and scarring of the kidneys that has already occurred [40,41]. These deficiencies are becoming more visible as newer medications have been introduced, namely, SGLT2 inhibitors and non-steroidal MRAs. As a result, both of these new agents provide tandem added benefit but still an incomplete benefit relative to RAAS blockade alone concerning kidney protection.

### 3.2. Sodium–Glucose Cotransporter 2 (SGLT2) Inhibitors

Recently, SGLT2 inhibitors have been proven to be the greatest advancement in treating DKD. Initially developed to lower blood glucose levels, SGLT2 inhibitors protect the kidneys by reducing sustained eGFR decline and delaying progression to kidney failure [8,42,43,44]. These results have been consistently replicated and confirmed through multiple double-blind randomized controlled trial findings [7,8,42,43]. There are many different mechanisms by which SGLT2 inhibitors protect the kidneys. They include both hemodynamic and non-hemodynamic pathways. SGLT2 inhibitors produce an effect on kidney function by blocking glucose and sodium reabsorption in the proximal tubule. This produces a negative feedback effect on the tubules and leads to the constriction of the afferent arterioles, resulting in a decrease in the amount of blood flow through the glomerular capillaries. This negative feedback effect counteracts the diabetic hyperfiltration effect and produces an initial decrease in eGFR that will be reversed over time and stabilize kidney function [45,46]. In addition, SGLT2 inhibitors reduce proximal tubular workload and oxygen consumption, thereby ameliorating renal cortical hypoxia, a key driver of tubulointerstitial injury in DKD [47].

Through additional mechanisms, SGLT2 inhibitors serve to protect the kidneys from damage by lowering both systemic and local (i.e., intrarenal) levels of inflammation, oxidative stress, and fibrotic signaling [48]. The effects of SGLT2 inhibitors on these pathways, along with the ability of SGLT2 inhibitors to lower blood pressure, weight, and uric acid levels, may be correlated with reduced apoptosis of proximal tubule cells and decreased levels of inflammatory cytokines produced by these cells [49].

Despite these substantial benefits, SGLT2 inhibitors reduce, but do not abolish, the risk of kidney disease progression in DKD. Across major outcome trials, including CREDENCE, DAPA-CKD, and EMPA-KIDNEY, relative risk reductions in primary renal endpoints ranged from approximately 30% to 40%, leaving a significant proportion of treated patients who continued to experience progressive eGFR decline or kidney failure [7,43]. These findings underscore the presence of considerable residual renal risk, even in patients receiving optimized therapy with RAAS blockade and SGLT2 inhibition.

Several factors contribute to the persistence of renal risk despite SGLT2 inhibitor therapy. First, the magnitude of benefit appears to vary across patient subgroups. SGLT2 inhibitors have shown effectiveness across multiple baseline eGFR and albuminuria levels. However, patients with substantial amounts of structural kidney damage, extensive scarring, and chronic diabetes have lower absolute benefits of SGLT2 inhibitors. Therefore, it seems likely that rather than having the ability to reverse the damage already caused in the kidneys by these diseases, SGLT2 inhibitors will primarily act to slow the rate at which the kidneys continue to be damaged [50]. While SGLT2 inhibitors improve the levels of albuminuria, the magnitude of reduction is smaller than that achieved with RAAS blockade. As a result, albuminuria will continue to be seen in the majority of cases treated with SGLT2 inhibitors [44].

Third, SGLT2 inhibitors primarily target hemodynamic and metabolic stress pathways and do not fully address other critical mechanisms of DKD progression, such as immune-mediated inflammation, maladaptive repair responses, and fibrotic remodeling. Post hoc analyses indicate that markers of inflammation and fibrosis may remain elevated in patients treated with SGLT2 inhibitors, potentially sustaining ongoing kidney damage [51]. This incomplete mechanistic coverage likely contributes to the continued eGFR decline in a subset of patients.

Another important limitation relates to treatment initiation and persistence in real-world practice. The introduction of SGLT2 inhibitors usually occurs later in the progression of DKD. At this point in DKD’s progression, there has been permanent structural damage to the kidneys, limiting the ability of the medications to have a significant impact on future kidney function. Additionally, patients may be discouraged from using SGLT2 inhibitors because of concerns about negative side effects (genital infections, volume depletion, euglycemic diabetic ketoacidosis in rare instances). These concerns may limit SGLT2 inhibitors’ use for frail or elderly patients, patients with advanced CKD stage 4 or 5, or patients with multiple other health problems [52,53]. Real-world data indicate that discontinuation rates remain non-negligible, further attenuating potential population-level benefits.

Notably, emerging evidence suggests that SGLT2 inhibitors may not uniformly protect against all forms of kidney injury. AKI risk appears reduced overall; however, susceptibility to hemodynamic AKI may persist in certain clinical contexts, such as acute illness, dehydration, or concomitant use of diuretics and RAAS inhibitors [19]. Recurrent subclinical AKI episodes may contribute to ongoing kidney damage and residual renal risk, even in patients receiving SGLT2 inhibitors [54].

While SGLT2 inhibitors provide the ability to delay the decline in kidney function due to DKD, these medications represent only one aspect of a holistic approach to DKD management. During clinical trials, eGFR will decrease for all subjects treated with SGLT2 inhibitors, but this will occur at a rate that is slower than before treatment with SGLT2 inhibitors. This reinforces the idea that SGLT2 inhibitors are an aspect of a comprehensive but complex approach to treating DKD rather than the sole route or approach to DKD control. Notably, several advantages associated with SGLT2 inhibitors have yet to be definitively validated. While several studies have demonstrated decreased levels of fibrotic and inflammatory biomarkers, these are generally modest to moderate reductions, and it is unclear whether these agents will ultimately eliminate the long-term structural damage caused by diabetes in the kidneys. Subgroup analyses have shown that participants with a significant amount of fibrosis (e.g., advanced degree) combined with drastically reduced renal function, as measured by eGFR < 15 mL/min/1.73 m^2^, and substantial nephron mass depletion (e.g., nephron loss over 50%) will not derive as much benefit as other participants receiving SGLT2 inhibitors. Overall, SGLT2 inhibitors meaningfully slow CKD progression but do not fundamentally alter late-stage disease biology.

### 3.3. Glucagon-like Peptide-1 Receptor Agonists (GLP-1 RAs)

GLP-1 RAs represent an integral part of the current mix of therapies available to treat type 2 diabetes, including people at high risk for cardiovascular disease. Initially offered only as a way to lower blood glucose, GLP-1 RAs have shown consistently greater benefit than might be anticipated based on their ability to control blood sugar. GLP-1 RAs have several positive effects on factors that can contribute to DKD, such as control of blood glucose levels, weight, blood pressure, and the presence of systemic inflammatory markers. Because of these multiple mechanisms of action, there is more interest in the possible use of GLP-1 RAs to provide kidney protection.

Several large trials examining cardiovascular outcomes with treatment of GLP-1 RAs have shown a lower risk of developing renal disease, specifically new cases of worsening albuminuria, in the group treated with GLP-1 RAs vs. placebo [55,56,57]. Three specific studies were conducted on the GLP-1 RAs liraglutide [LEADER], semaglutide [SUSTAIN-6], and dulaglutide [REWIND], where significant differences in the rate of progression from normal to abnormal urinary protein levels were found in all cases. A recent meta-analysis confirmed that these medications significantly reduced the combined renal event of albuminuria but demonstrated neutral or inconsistent effects on hard renal outcomes such as sustained eGFR decline or progression to kidney failure, highlighting important uncertainty regarding their role as primary renoprotective agents [58].

The renal effects of GLP-1 RAs appear to be mediated predominantly through indirect and pleiotropic mechanisms rather than direct hemodynamic actions on the glomerulus. The metabolic control, decreased insulin resistance, and increased weight loss associated with GLP-1 RA treatment reduce renal metabolic stress. In addition, results from experimental and translational studies show that through activation of GLP-1 receptors, anti-inflammatory and antioxidant effects are exerted on the kidney, including the down-regulation of pro-inflammatory cytokines, blocking of nuclear factor kappa-light-chain-enhancer of activated B cells (NF-κB) pathways, and a decrease in oxidative stress [59,60]. GLP-1 RAs have additionally demonstrated benefits to the endothelium and the ability to decrease vascular stiffness. Therefore, GLP-1 RAs can potentially reduce intrarenal microvascular injury as a contributor to the progression of DKD [60].

Despite these favorable effects, several limitations constrain the ability of GLP-1 RAs to substantially reduce residual renal risk. First, the renal benefits observed in major outcome trials are largely confined to albuminuria reduction, while effects on eGFR slope and kidney failure outcomes are comparatively small or statistically nonsignificant. This contrasts with SGLT2 inhibitors, which consistently slow eGFR decline and reduce kidney failure risk, and suggests that GLP-1 RAs may primarily modify early glomerular injury rather than advanced structural damage [48,58].

Second, GLP-1 RAs do not address the main causes of DKD progression, namely tubulointerstitial fibrosis, maladaptive repair responses, and irreversible nephron loss. Although there have been anti-inflammatory effects with GLP-1 RAs, they may not be adequate to stop progression once a person has established fibrotic remodeling. This is especially true for patients with advanced CKD, as their structural kidney damage would likely be minimally impacted by anti-inflammatory medication and metabolic modification [61].

Third, the evidence base for GLP-1 RAs in patients with advanced CKD remains limited. Many cardiovascular outcome trials excluded patients with severely reduced eGFR, and although newer agents appear safe across a broad range of kidney function, robust data on renal outcomes in advanced DKD are still emerging [2]. Gastrointestinal adverse effects, including nausea and vomiting, may further limit tolerability and long-term adherence, particularly in older or frail patients with CKD [3,4].

One additional factor to think about is that GLP-1 RAs are generally initiated later in the disease process (most frequently, after the development of albuminuria or worsening of eGFR). As seen with other medications, starting treatment late may reduce the true renoprotective effect of these medications and, therefore, may leave the patient with an ongoing risk for residual renal injury. Furthermore, many studies looking at the real-life effects of GLP-1 RAs demonstrate variability in how they are prescribed or used by patients based on factors such as cost, route of administration, and willingness to use the drug.

Recent data from dedicated kidney outcome trials, such as the FLOW study [62] evaluating semaglutide in patients with DKD, suggest that GLP-1 RAs may confer more robust renal protection than previously appreciated, including slowing of eGFR decline. However, even in these contemporary studies, renal risk is reduced rather than eliminated, reinforcing the concept that GLP-1 RAs, while beneficial, do not fully prevent DKD progression.

### 3.4. Non-Steroidal Mineralocorticoid Receptor Antagonists (nsMRAs)

The nsMRAs offer new treatment possibilities for patients with DKD by blocking the excess activity of the MRs, which leads to inflammation and scarring of the kidneys. While the current medications used to treat DKD (ACE-I/ARBs and SGLT2 inhibitors) lower the incidence of loss of kidney function, they do not completely prevent or manage the damage caused by excess MR activity. Persistent MR activation represents a key non-hemodynamic driver of residual renal risk, operating downstream of blood pressure and glycemic control. The best-studied nsMRA is finerenone, which has demonstrated the best clinical evidence of improved renal and cardiovascular outcomes. Mechanistically, MR activation in the kidney promotes macrophage and T-cell recruitment, oxidative stress, endothelial dysfunction, and profibrotic signaling that culminate in tubulointerstitial fibrosis and progressive nephron loss. By antagonizing MR activity, finerenone is proposed to mitigate these downstream inflammatory and fibrotic cascades, thereby addressing a dimension of DKD pathobiology that persists despite hemodynamic optimization and improved glycemic control [63,64]. At the molecular level, finerenone exerts distinct transcriptional effects compared with steroidal MRAs by inhibiting serum/glucocorticoid-regulated kinase 1 (SGK1) signaling and preventing maladaptive MR–cofactor recruitment, resulting in more selective suppression of pro-inflammatory and pro-fibrotic gene expression [65].

The clinical efficacy of finerenone in DKD is anchored by two pivotal, placebo-controlled outcome trials conducted on top of maximally tolerated RAAS blockade. In FIDELIO-DKD, finerenone reduced the risk of CKD progression (a composite that included kidney failure and sustained eGFR decline) and also lowered cardiovascular events in a population enriched for more advanced CKD and higher albuminuria, reinforcing the concept that MR-driven pathways remain clinically relevant even under optimized background therapy [10]. In FIGARO-DKD, which enrolled a broader DKD spectrum including earlier CKD stages and/or moderately increased albuminuria, finerenone significantly improved cardiovascular outcomes, with supportive, though comparatively less prominent, effects on kidney endpoints, suggesting that the balance of benefit may vary by baseline phenotype and event propensity [11]. A prespecified pooled analysis (FIDELITY) integrating both trials strengthened the evidence base by demonstrating consistent reductions in both cardiovascular and kidney outcomes across the combined population, supporting finerenone as a risk-reducing add-on therapy in DKD rather than a niche intervention confined to a single disease stage [66].

More recently, real-world comparative effectiveness data have expanded this evidence base by directly contrasting finerenone with steroidal MRAs. In a large, international target trial emulation using electronic health record data from the TriNetX network, finerenone was compared head-to-head with spironolactone in patients with CKD and type 2 diabetes receiving contemporary background therapy. After propensity score matching, finerenone was associated with significantly lower risks of major adverse kidney events, major adverse cardiovascular events, and all-cause mortality over a median follow-up of 1.3 years. Notably, the magnitude of mortality reduction translated into a low number needed to treat, underscoring the clinical relevance of MR-selective blockade even over relatively short follow-up periods. These findings support the concept that nsMRAs may provide superior cardiorenal protection compared with traditional steroidal MRAs in routine clinical practice, beyond differences attributable solely to blood pressure or albuminuria reduction [65].

These data have translated rapidly into guidance documents and layered-treatment algorithms. The KDIGO 2022 guideline for diabetes management in CKD explicitly recognizes finerenone as the nsMRA with proven kidney and cardiovascular benefits and recommends considering an nsMRA for patients with type 2 diabetes, CKD, and persistent albuminuria despite foundational therapy, taking into account an acceptable serum potassium level and the ability to monitor it over time [64]. This positioning is clinically meaningful in the context of residual renal risk: finerenone can be added when “standard” risk reduction (RAAS blockade ± SGLT2 inhibition and risk-factor control) does not sufficiently suppress ongoing progression risk.

Despite these advances, nsMRAs also illustrate why residual renal risk persists even in the era of multidrug renoprotection. First, the strongest evidence for finerenone is in albuminuric DKD, because pivotal trials enrolled patients with persistent albuminuria and mandated RAAS inhibitor background therapy. Thus, generalizability to non-albuminuric DKD (a growing clinical phenotype) is less direct and remains a key evidence gap. Second, hyperkalemia risk, while generally more manageable than with steroidal MRAs in many contexts, remains the dominant implementation barrier, particularly in patients with lower eGFR, higher baseline potassium, concomitant RAAS blockade, or intercurrent illness. In both FIDELIO-DKD and FIGARO-DKD, hyperkalemia events were more frequent with finerenone than placebo, necessitating structured monitoring and dose adjustments that can be challenging outside trial settings [10,11]. However, comparative real-world data indicate that finerenone is associated with a substantially lower incidence of treatment-emergent hyperkalemia than spironolactone, including severe hyperkalemia, which may translate into improved treatment persistence and more sustained MR blockade in practice [65]. Third, while finerenone targets inflammatory and fibrotic signaling, its benefits are additive rather than curative: patients continue to experience progression events over time, consistent with the reality that established nephron loss and advanced structural remodeling may be only partially modifiable, even with therapies that address fibrosis-related biology [66]. Finally, as with other DKD therapies, the real-world impact of nsMRAs is likely influenced by therapeutic inertia, monitoring burden, and the clinical complexity of patients most likely to benefit. These implementation factors may attenuate population-level effectiveness and contribute to the persistence of residual renal risk despite mechanistically targeted intervention. Despite favorable trial results, nsMRAs do not eliminate progression events, and their benefits appear additive rather than transformative, particularly in patients with advanced structural kidney damage, emphasizing that MR blockade modifies, but does not reverse, established disease trajectories.

### 3.5. Evidence of Persistent Progression Despite Optimal Treatment

Despite the availability of modern “ideal” multi-drug treatments for DKD, including an optimal combination of maximum tolerated levels of all RAAS-blocking agents and SGLT2 inhibitors, many patients with DKD experience progressive renal decline. The results of multiple independent and high-level RCTs show that the overall relative risk for kidney outcome events has decreased significantly. These studies show that a significant proportion of patients with DKD still experience unacceptably high rates of residual renal risk. The biological processes causing residual renal risk are not well established. However, persistent inflammation, fibrogenic pathways, tubulointerstitial damage, and metabolic memory represent potential targets for further evaluation. Many of these hypotheses are based mostly on post hoc analyses from RCTs, observational cohort studies, biomarker studies, and experimental studies. Therefore, while the available evidence supports the theories of continued residual renal risk, they are biological hypotheses supported by data rather than direct or conclusive evidence of the cause-and-effect relationship.

Canagliflozin (CREDENCE trial) has been shown to reduce the relative risk of a composite kidney outcome among people with established nephropathy and on ACE-I or ARB therapy. However, the rate of occurrence of kidney events on active treatment in this study was 43.2 per 1000 patient-years, indicating that there remains an increased risk for clinically relevant progressions, despite having received treatment (compared to a rate of 61.2 per 1000 patient-years on placebo) [43]. Similar findings were reported in the DAPA-CKD study. Dapagliflozin lowered the odds of developing a sustained 50% or greater decrease in eGFR, kidney failure, and renal death. However, the incidence of renal events occurred during the entire follow-up period, indicating that SGLT2 inhibitors will not halt the progression of DKD but rather slow progression [44]. The continuing reasons for renal event occurrence were reaffirmed by the EMPA-KIDNEY study’s median follow-up of approximately two years, with 13.1% of patients receiving empagliflozin developing progression of kidney disease or cardiovascular death, versus 16.9% of patients in the comparator (placebo) group. As such, these studies highlight that SGLT2 inhibitors have a significant benefit and can slow the progression of DKD. However, DKD progression will occur and is a risk factor for kidney failure [8].

Persistent progression is also observed when targeting inflammatory and fibrotic pathways beyond hemodynamic control. In FIDELIO-DKD, conducted on a background of optimized RAAS blockade, the primary kidney outcome still occurred in 17.8% of patients treated with finerenone over a median follow-up of 2.6 years, compared with 21.1% in the placebo group [10]. The pooled FIDELITY analysis, integrating data from FIDELIO-DKD and FIGARO-DKD, reinforced this observation at scale: although finerenone reduced both kidney and cardiovascular outcomes across a broad DKD spectrum, event rates remained substantial, consistent with ongoing disease activity and irreversible structural damage in many patients [66].

More recent evidence from incretin-based therapy further supports the concept of residual renal risk despite therapeutic layering. In the FLOW trial, semaglutide significantly reduced major kidney disease events in patients with DKD; nevertheless, 331 primary renal events still occurred in the semaglutide group during follow-up (compared with 410 in the placebo group), indicating that even newer agents with favorable effects on eGFR slope and hard renal endpoints do not fully prevent progression once DKD is established [62].

The major renoprotective therapies and their limitations contributing to residual renal risk are summarized in Table 1. Beyond summarizing therapeutic classes, Table 1 highlights that residual renal risk arises from shared limitations across drug classes, namely incomplete coverage of inflammation, fibrosis, tubulointerstitial injury, and structural irreversibility, thereby emphasizing common biological constraints rather than drug-specific shortcomings.

Overall, the pooled data demonstrate a consistent renoprotective effect of modern therapeutics by decreasing relative risk in all available randomized trials. However, there are significant limitations that affect the interpretation of these data. The nature of the patient populations in RCTs (i.e., patients enrolled in controlled trials are selected for entry into the study based upon what is known about their acute or chronic kidney disease and for having been diagnosed with albuminuria) limits the applicability to real-world patients who are seen in a clinical setting with varying comorbidities. Some studies have examined similar patient populations to the cohort seen in RCTs. However, these studies are not randomized, and therefore, there is the potential for confounding and survivor bias. Furthermore, while maximum relative risk reduction has been the focus of published studies, the absolute event rates and continued eGFR decline that have been reported in studies indicate that all available therapies only provide partial success in achieving their objective, and no therapy is considered curative. Head-to-head comparisons of drug classes through testing have been conducted by a small number of studies only, and there are primarily indirect findings supporting optimal sequenced/combinations of drugs. Therefore, based on the conclusion of this study, we see that the continued renal risk being observed in these studies is not only due to poor compliance, but also to the underlying biological factors being encountered, which cannot be corrected by the available treatments at this time.

## 4. Mechanisms Underlying Residual Renal Risk

The following mechanistic pathways are supported by a combination of experimental data, observational studies, and post hoc clinical analyses. However, direct interventional evidence demonstrating that selective modulation of these pathways improves hard renal outcomes remains limited.

### 4.1. Persistent Inflammation

Having established the persistence of residual renal risk despite contemporary therapy, this section shifts from descriptive outcomes to a mechanistic explanation. Although multiple mechanisms have been implicated in the persistence of kidney disease progression despite contemporary therapy, the relative contribution of individual pathways remains incompletely defined. Evidence from experimental models, biomarker studies, and post hoc clinical analyses suggests that persistent inflammation, fibrosis, tubulointerstitial and microvascular injury, and metabolic memory each contribute to residual renal risk. However, the strength of evidence supporting these mechanisms varies substantially.

Chronic inflammation results in lasting detrimental effects on kidney health and remains a key factor in the development of DKD among patients being treated according to current clinical guidelines. Much of the clinical evidence linking inflammation to renal outcomes is associative rather than causal, derived from observational studies and biomarker analyses rather than intervention trials directly targeting inflammatory pathways. This distinction is important when interpreting inflammation as both a driver of disease progression and a potential therapeutic target. Research suggests that all the aforementioned therapies (RAAS blockade, SGLT2 inhibitors, GLP-1 RAs, and nsMRAs) have some effect on reducing kidney inflammation, but none are sufficient to eliminate the ongoing kidney damage from DKD. Therefore, inflammation continues even after optimal treatment, resulting in continued structural and functional loss of nephrons.

DKD causes chronic inflammation through a variety of systemic factors and processes that interact within the kidney. Diabetes mellitus leads to chronic hyperglycemia, which contributes to the development of DKD through multiple pathogenic mechanisms, including oxidative stress, accumulation of advanced glycation end products (AGEs), and disturbances in lipid metabolism. It also caused both primary (innate) and secondary (adaptive) immune responses related to the kidneys due to the increases in pro-inflammatory mediators [cytokines including tumor necrosis factor-alpha (TNF-α); interleukin-1 beta (IL-1β); IL-6; monocyte chemoattractant protein-1 (MCP-1)] [15,67,68]. This increase in pro-inflammatory mediators in the kidneys leads to recruitment of immune cells (macrophages and T lymphocytes) into the kidneys. Immune cells are located in both the glomerular and tubulointerstitial regions of inflammation, providing an enhancement of the responses through feedback on local pro-inflammatory signaling. Consequently, the presence of inflammatory cells surrounding renal tubules directly correlates with acute tubular injury and an increased fibrotic response in the renal interstitium. These two types of injury can be more informative indicators of decline in renal function than the presence of glomerular lesions alone [69,70].

Despite conventional treatment, individuals with DKD show elevated inflammatory markers. The presence of elevated levels of TNF receptor 1 (TNFR1) and TNF receptor 2 (TNFR2), C-reactive protein (CRP), and several other inflammatory cytokines has been described as predictive for an individual’s increased risk of progressive development of kidney failure and diminished eGFR independent of albuminuria or traditional risk factors [54,71,72]. In addition, even following treatment with either RAAS inhibitors or SGLT2 inhibitors, patients usually continue to have declining eGFR levels as a result of the actions of inflammation, revealing that these currently available therapies do not eradicate the influence of inflammatory cytokines on renal function and damage.

Several mechanisms explain why inflammation remains active despite contemporary treatment. The majority of anti-inflammatory benefits provided by therapies already available are mediated or indirect. Therefore, although these interventions primarily improve hemodynamic parameters, metabolic control, and/or neurohormonal signaling pathways, they do not directly suppress or modulate immune pathway activation. Although sodium–glucose cotransporter 2 inhibitors and glucagon-like peptide-1 receptor agonists exert anti-inflammatory effects, these effects are relatively limited, particularly in individuals with long-standing chronic disease or established tubulointerstitial damage [48,51,59]. Second, intrarenal inflammation may become self-sustaining through local cytokine networks, hypoxia, and maladaptive repair responses, rendering it less responsive to systemic interventions introduced later in the disease course [14].

In addition, the phenomenon of “metabolic memory” contributes to persistent inflammation in DKD. Epigenetic modifications induced by prior hyperglycemic exposure can result in long-lasting activation of pro-inflammatory genes, even after glycemic control improves [23,24]. This effect provides a mechanistic explanation for why inflammatory signaling and kidney damage may continue despite subsequent optimization of glucose levels and renoprotective therapy.

The persistent inflammatory response continues to be relevant to clinical practice based on the limited data supporting the efficacy of current anti-inflammatory therapies. Broad anti-inflammatory strategies have failed to achieve the expected outcome as many patients can develop unacceptable toxicity due to those strategies [40]. Ongoing efforts continue to develop new agents aimed at a variety of specific cytokines and chemokines with respect to their effects through various metabolic and/or hemodynamic pathways that lead to inflammatory development. The maintained presence of inflammatory biomarkers and the decreasing renal function in treated individuals demonstrate that there is an urgent need for further refined anti-inflammatory strategies that are based on distinct phenotypes.

### 4.2. Fibrosis and Structural Irreversibility

Progressive renal fibrosis represents a central and largely irreversible mechanism underlying residual renal risk in DKD. While contemporary therapies effectively attenuate hemodynamic stress, metabolic injury, and, in part, inflammation, they are substantially less effective at reversing established structural damage. Once fibrotic remodeling of the kidney parenchyma is present, particularly within the tubulointerstitial compartment, disease progression often continues despite optimal treatment, reflecting a critical “point of no return” in DKD pathophysiology.

Excessive buildup of extracellular matrix (ECM) components such as collagen types I and III, fibronectin, and laminin disrupts normal tissue architecture, decreases capillary density (rarefaction), and causes progressive nephron loss [14,73]. Although there are early fibrotic changes that can be dynamic and modifiable, by the time advanced fibrosis is reached, it is generally accepted that advanced fibrosis is biologically stable and not reversible. It has been shown in many histopathological studies that interstitial fibrosis and tubular atrophy are much stronger predictors of long-term decline in renal function than glomerular lesions alone, which emphasizes the importance of structural remodeling in determining prognosis [69,74].

It is noteworthy that fibrotic remodeling will continue even when upstream triggers (e.g., hyperglycemia and intraglomerular hypertension) have been partially treated. This represents the continuing cycle of development of renal fibrosis through a continuous supply of factors, including activated myofibroblasts, maladaptive epithelial–mesenchymal signaling, and chronic deposition of ECM components that can cause renal injury without reliance on the initial noxious stimuli [75,76]. The profibrotic factors [e.g., TGF-β, connective tissue growth factor (CTGF), and Wingless-related integration site/beta-catenin signaling pathway (Wnt/β-catenin) pathway] also remain active during the late stages of DKD, resulting in ongoing accumulation of ECM and inhibition of normal repair processes [77,78].

Current renoprotective therapies only partially address fibrotic pathways. RAAS blockade reduces fibrogenic signaling indirectly by lowering intraglomerular pressure and attenuating angiotensin II-mediated TGF-β activation, but does not eliminate fibrotic progression, particularly in advanced disease [79]. SGLT2 inhibitors appear to slow fibrotic remodeling through improvements in tubular oxygenation, metabolic efficiency, and inflammation. However, experimental and clinical data suggest that these effects primarily delay rather than reverse established fibrosis [46,51]. Similarly, nsMRAs such as finerenone directly target profibrotic MR signaling and reduce progression risk, yet residual fibrotic burden persists, as evidenced by continued event accrual in outcome trials [10,66].

A critical clinical implication of fibrosis-related irreversibility is the timing of therapeutic intervention. Many patients initiate renoprotective therapy only after significant structural damage has already occurred, limiting the potential for disease modification. Observational data and post hoc trial analyses consistently show that patients with more advanced CKD, greater degrees of fibrosis, or longer disease duration derive smaller absolute renal benefits from therapy compared with those treated earlier in the disease course [50,80]. This highlights fibrosis not only as a mechanism of residual risk but also as a determinant of therapeutic responsiveness.

Furthermore, there is a lack of validated, noninvasive tools to accurately measure renal fibrosis. While a kidney biopsy is the gold standard for measuring the fibrotic burden, the invasive procedure does not allow for widespread application. As such, clinicians typically depend upon surrogate markers like a decrease in eGFR and proteinuria, which may not accurately represent the level of irreversible structural damage [81]. This diagnostic gap hampers risk stratification and may lead to overestimation of the potential benefit of therapy in patients with advanced fibrotic disease.

Attempts to directly target renal fibrosis have thus far yielded limited clinical success. Multiple antifibrotic agents have failed to demonstrate meaningful benefit in DKD, likely due to disease heterogeneity, late intervention, and the complexity of fibrotic signaling networks [33]. These failures reinforce the concept that fibrosis represents a final common pathway of injury that is difficult to reverse once established, even when upstream pathogenic processes are addressed.

While fibrosis is considered to play a major role in DKD progression, many important gaps still exist regarding the role of connective tissue in DKD. Most human studies that established relationships between the amount of fibrosis and the development of DKD are based on studies that used samples of biopsy tissue, which limits the understanding of how fibrosis develops over time and how patients may respond to pharmacologic treatment. The failure of antifibrotic medications during clinical trials raises several unresolved questions regarding whether the development of fibrosis will cause DKD to worsen or if fibrosis is only a marker of already existing, irreversible damage to the kidneys. These unresolved questions prevent the development of effective therapies to prevent the progression of DKD and require clinicians to intervene earlier in the disease course before the development of fibrosis becomes genetically or biologically hardwired.

### 4.3. Tubulointerstitial and Microvascular Injury

Tubulointerstitial and microvascular injury constitute closely interconnected mechanisms that play a pivotal role in the persistence of renal risk in DKD, particularly in patients receiving contemporary renoprotective therapy. While traditional models of DKD emphasized glomerular injury as the primary driver of progression, accumulating evidence indicates that damage to the tubular compartment and the intrarenal microvasculature is a dominant determinant of long-term kidney function decline and an important contributor to residual renal risk despite optimal treatment.

During the early stages of DKD, tubular injury is already present alongside multiple hemodynamic and metabolic stressors, including hyperglycemia, increased proximal tubular reabsorption of glucose and sodium, oxidative stress, and exposure to filtered proteins. Because proximal tubular epithelial cells have high energy demands and rely heavily on oxidative metabolism for ATP generation, they are particularly vulnerable to injury. This susceptibility exists independently of, and may precede, overt glomerular damage during the course of DKD. Therefore, sustained exposure of tubular cells to supraphysiological levels of metabolic and hemodynamic stress leads to progressive cellular dysfunction, mitochondrial impairment, defective fatty acid β-oxidation, and activation of pro-inflammatory and pro-fibrotic signaling pathways. Additionally, tubular cell injury can occur independent of or before any glomerular injury, which creates a basis for non-albuminuric DKD as a distinct phenotype for continued progressive kidney damage [2,82].

The injury to the microvasculature ultimately exacerbates tubulointerstitial damage and makes the disease irreversible. Several factors, including diabetes and associated endothelial dysfunction, have been documented as being able to cause kidney disease. Diabetes-induced capillary basement membrane thickening as well as progressive rarefaction of the peritubular capillaries contribute to decreased oxygen delivery to the kidneys and chronic renal hypoxia [83,84]. The effect of hypoxia in promoting tubular cell apoptosis/injury, inflammatory responses, and fibrotic remodeling has been attributed to activation of the hypoxia-inducible pathway via the initiation of renal cell injury, thus creating a cycle of microvascular rarefaction and hypoxia that continues to result in renal damage, even when systemic risk factors have been effectively managed.

Although current therapies provide partial benefit, they do not fully reverse or repair established damage to the renal tubules, interstitium, or microvascular architecture [79]. While the use of agents that inhibit the RAAS reduces pressure on the kidneys and decreases the amount of proteinuria, these therapies do not address how the kidneys metabolize and utilize nutrients or how they function overall. Conversely, SGLT2 inhibitors enhance the communication of both tubule and glomerulus feedback loops and allow more efficient oxygen delivery to kidney cells, thereby improving their ability to metabolize and utilize glucose and other substances. Yet again, these types of medications only reverse damage after severe or progressive disease has occurred [46,47]. Additionally, nsMRAs reduce the inflammatory processes and fibrosis associated with CKD. They do not directly re-establish blood flow or build tubular structures in the kidneys [51].

Studies that use clinical or translational methods have demonstrated that tubulointerstitial/microvascular issues have prognostic value. Most histopathological studies have established that tubular atrophy, interstitial fibrosis, and peritubular capillary loss are the strongest predictors of eGFR decline and kidney failure when compared to glomerular pathology [69]. In addition to this, the majority of patients will continue to have elevated levels of circulating/urinary indicators of tubular injury [e.g., kidney injury molecule-1 (KIM-1), neutrophil gelatinase-associated lipocalin (NGAL), and other indicators of endothelial dysfunction] after being treated with appropriate management options. These indicators have also proved to be predictive of additional adverse renal outcomes independently of the eGFR [54,85].

An additional challenge is that tubulointerstitial and microvascular injury are often clinically silent and poorly captured by conventional markers such as albuminuria and eGFR. As a result, substantial structural damage may accumulate before progression is recognized, limiting the window for effective intervention. This diagnostic gap contributes to delayed therapy initiation and overestimation of treatment efficacy in patients with advanced, structurally driven disease [80].

### 4.4. Metabolic Memory and Prior Hyperglycemic Exposure

The concept of metabolic memory, also referred to as the “legacy effect”, represents a critical mechanism underlying residual renal risk in DKD. It describes the phenomenon whereby prior periods of poor glycemic control exert long-lasting deleterious effects on renal structure and function that persist despite subsequent optimization of blood glucose levels and implementation of contemporary renoprotective therapies. This mechanism provides a compelling explanation for continued kidney disease progression in patients who appear to be well-treated according to current standards.

The existence of metabolic memory was first established in large, long-term clinical trials of diabetes management. In both the Diabetes Control and Complications Trial (DCCT) and its observational follow-up (EDIC), early intensive glycemic control resulted in a durable protection against microvascular complications, including nephropathy, long after glycemic differences between treatment groups had converged [86,87]. Similar observations were reported in the UK Prospective Diabetes Study (UKPDS), where early glucose-lowering benefits translated into sustained reductions in microvascular outcomes over extended follow-up [88]. These findings indicate that early metabolic insults imprint long-term pathological changes that are not readily reversible by later glycemic improvement.

At the molecular level, metabolic memory is mediated by persistent alterations in cellular signaling, gene expression, and chromatin structure induced by hyperglycemia. These changes include sustained activation of oxidative stress pathways, accumulation of AGEs, and stable epigenetic modifications affecting inflammatory and profibrotic gene networks [88,89,90]. In renal cells, including podocytes, mesangial cells, and tubular epithelial cells, hyperglycemia-induced epigenetic marks, such as DNA methylation, histone modifications, and non-coding RNA expression, can maintain a pro-inflammatory and pro-fibrotic transcriptional profile even after normalization of glucose levels [24,91].

Oxidative stress plays a central role in linking hyperglycemic exposure to long-term renal injury. Excess glucose flux through mitochondrial pathways increases reactive oxygen species production, which in turn activates stress-sensitive signaling cascades involving nuclear factor-κB, protein kinase C, and TGF-β [15,92]. Importantly, oxidative stress–induced damage may persist through self-amplifying feedback loops, reinforcing inflammatory and fibrotic signaling independently of ongoing hyperglycemia. This mechanism helps explain why modern therapies that effectively lower glucose and improve hemodynamics do not fully suppress progression in patients with long-standing diabetes.

Clinically, markers of prior glycemic burden have been shown to predict renal outcomes independently of current glycemic control. Long-term glycated hemoglobin exposure, glycemic variability, and duration of diabetes are all associated with faster eGFR decline and increased risk of kidney failure [22,54,72]. These observations underscore that residual renal risk reflects cumulative metabolic injury rather than solely current disease activity.

Metabolic memory also interacts with other mechanisms of residual risk, including inflammation, fibrosis, and microvascular injury. Hyperglycemia-induced epigenetic changes can sensitize renal cells to inflammatory stimuli, amplify cytokine responses, and promote maladaptive repair following AKI or hemodynamic stress. As a result, patients with extensive prior hyperglycemic exposure may exhibit heightened vulnerability to progressive kidney damage, even when treated with agents that target downstream pathways such as RAAS activation or SGLT2 inhibition.

Importantly, current therapies only partially address metabolic memory. While early initiation of SGLT2 inhibitors, GLP-1 Ras, and optimized glucose control may attenuate the development of new epigenetic injury, there is limited evidence that established metabolic memory can be reversed. This limitation has important implications for clinical practice, emphasizing the need for early, aggressive metabolic control and timely initiation of renoprotective therapy to prevent irreversible molecular and structural changes before they become self-sustaining [40,80].

**Figure 1:**
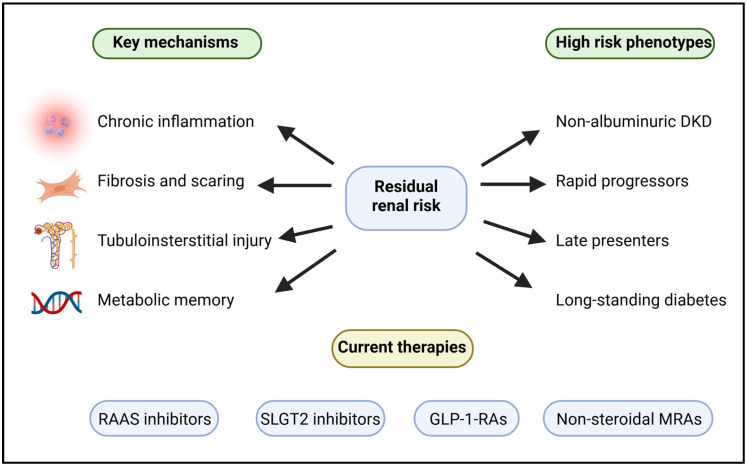
Conceptual framework of residual renal risk in diabetic kidney disease (DKD). This schematic illustrates the multifactorial mechanisms that contribute to persistent kidney disease progression in DKD despite guideline-directed, multidrug therapy. Persistent intrarenal inflammation, progressive fibrosis with structural irreversibility, tubulointerstitial and microvascular injury, and metabolic memory induced by prior hyperglycemic exposure interact to sustain ongoing renal damage and nephron loss. Renoprotective therapies, including renin-angiotensin–aldosterone system blockade, sodium-glucose cotransporter 2 inhibitors, glucagon-like peptide-1 receptor agonists, and non-steroidal mineralocorticoid receptor antagonists, attenuate but do not fully suppress these pathogenic pathways. The convergence of these mechanisms results in residual renal risk, manifesting clinically as continued estimated glomerular filtration rate decline and progression toward kidney failure in susceptible patient phenotypes.

The key pathogenic mechanisms that sustain residual renal risk in DKD are summarized in Figure 1. Figure 1 is intended as an integrative framework that synthesizes clinical trial evidence, mechanistic pathways, and disease irreversibility, illustrating how multiple partially treated processes converge to sustain kidney function decline despite optimized therapy. Importantly, several mechanistically compelling targets, particularly antifibrotic and broad anti-inflammatory strategies, have failed to demonstrate consistent clinical benefit in trials to date, underscoring the gap between biological plausibility and therapeutic efficacy in DKD.

## 5. Clinical Phenotypes at High Residual Renal Risk

Despite the availability of renoprotective therapies, DKD remains a heterogeneous condition with marked interindividual variability in progression and treatment response. Rather than revisiting the concept of residual renal risk itself, this section applies the established framework to identify clinical phenotypes in which residual renal risk is concentrated and most clinically consequential. Accumulating evidence indicates that residual renal risk is concentrated in distinct clinical phenotypes rather than being uniformly distributed across the DKD population. These phenotypes reflect differences in underlying pathophysiology, disease stage at treatment initiation, cumulative metabolic burden, and comorbidity profile, all of which influence the persistence of kidney function decline despite guideline-directed care.

One prominent phenotype associated with high residual renal risk is non-albuminuric DKD, characterized by reduced eGFR in the absence of significant albuminuria. This phenotype has become increasingly prevalent in recent decades, accounting for up to 30–40% of DKD cases in contemporary cohorts, likely due to earlier diabetes diagnosis, widespread use of RAAS inhibitors, and improved glycemic control [2,17]. Although initially considered relatively benign, non-albuminuric DKD is now recognized to carry a substantial risk of progressive eGFR decline and cardiovascular morbidity [93,94]. Pathophysiologically, this phenotype appears to be driven predominantly by tubulointerstitial injury, microvascular disease, and ischemic mechanisms rather than classic glomerular hyperfiltration and proteinuric injury, rendering therapies that primarily target albuminuria and intraglomerular pressure less effective and contributing to persistent progression [95,96].

Another high-risk group comprises patients who exhibit rapid kidney function decline despite apparent optimal therapy. These so-called rapid progressors experience accelerated eGFR loss that exceeds expected age-related decline and remain at high risk for kidney failure even when treated with RAAS blockade, SGLT2 inhibitors, and other agents [97]. Rapid progression has been linked to a high inflammatory burden, recurrent episodes of AKI, persistent metabolic stress, and extensive baseline tubulointerstitial fibrosis [54,71]. Importantly, this phenotype may be under-recognized in routine practice, as short-term stabilization of albuminuria or transient eGFR plateaus can obscure ongoing structural injury, highlighting the limitations of conventional markers for assessing residual risk [29].

Delayed initiation of renoprotective therapy represents another major contributor to high residual renal risk. Patients who commence contemporary treatment at advanced stages of CKD frequently have extensive irreversible structural damage, including nephron loss, interstitial fibrosis, and microvascular rarefaction, which constrains the capacity of therapy to meaningfully alter disease trajectory [50,69]. Post hoc analyses of major outcome trials consistently show that while relative risk reductions with SGLT2 inhibitors or nsMRAs are preserved across CKD stages, absolute renal benefits are attenuated in advanced disease, and progressive eGFR decline is observed [7,66]. This phenotype underscores the critical importance of early detection and timely intervention to limit irreversible injury.

Despite multiple-drug treatments based on clinical guidelines aimed at maintaining kidney function by maximizing RAAS inhibitors, SGLT2 inhibitors, GLP-1-Ras, and nsMRAs, there are still significant numbers of DKD patients whose kidney function continues to deteriorate. These so-called therapeutic “non-responders” have an ongoing decline in eGFR, due to tubulointerstitial injury, microvascular rarefaction, inflammation, and irreversible fibrotic remodeling, processes that are only partially modulated by current therapies and may occur independently of albuminuria dynamics [8,10,43,44,66,74,80]. While it has previously been thought that an improvement in albuminuria is equivalent to the arrest of disease, when a patient’s eGFR drops lower than previously thought because they have had the recommended medical interventions progression, continued declines in eGFR despite an apparent biochemical improvement indicate that a patient may be classified as a non-responder [17,93,94,95,96]. Identifying a non-responder to therapeutic interventions as a separate clinical entity has important implications for both clinical practitioners and researchers in developing guidelines for the management of CKD. The identification of this distinct clinical entity underscores that longitudinal evaluation of eGFR, rather than assessment of eGFR based on short-term surrogate markers only, should be performed. In addition, the identification of patients with disproportionate structural injury and the establishment of pragmatic treatment goals to facilitate the slow-down of the progression of the disease (as opposed to making it return to baseline) after irreversible damage are critical issues.

Patients with long-standing diabetes and high cumulative metabolic burden also represent a subgroup with elevated residual renal risk. Independent of current glycemic control, longer diabetes duration and historical hyperglycemic exposure are strongly associated with faster eGFR decline and increased risk of kidney failure, reflecting the effects of metabolic memory and persistent molecular and epigenetic alterations [22,72]. In these patients, therapies may slow progression but are often insufficient to fully overcome structural and cellular injury.

Finally, DKD patients with significant multimorbidity, including advanced age, cardiovascular disease, heart failure, and frailty, constitute a phenotype in which residual renal risk is amplified by competing pathophysiological processes and therapeutic constraints. Hemodynamic instability, polypharmacy, intolerance to full-dose renoprotective agents, and frequent intercurrent illness often limit treatment optimization in this population, leading to continued kidney function decline despite best available care [98].

Key mechanisms and clinical phenotypes associated with high residual renal risk are summarized in Table 2. Table 2 links mechanistic pathways to specific high-risk clinical phenotypes, thereby providing a translational bridge between biological processes and patient-level risk stratification, rather than a simple catalog of mechanisms or phenotypes.

## 6. Therapeutic Gaps and Future Directions

The following discussion integrates the preceding evidence, mechanistic insights, and phenotypic distinctions to identify therapeutic gaps, rather than restating the concept of residual renal risk itself. There are several important challenges in interpreting the current evidence base for residual risks of renal impairment, based on the lack of a clearly defined hierarchy among the various therapeutic mechanisms and clinical outcomes. RCTs support the clinical efficacy of all drug classes, although they do not indicate which pathways may have the most significant clinical actionability for patients or which patient populations may benefit the most from additional interventions augmenting these pathways. In contrast, observational studies and mechanistic hypotheses identify many potential target pathways. Thus, they serve as evidence supporting hypotheses, rather than providing definitive answers to unanswered questions. Consequently, many proposed strategies to mitigate or eliminate residual renal impairment risk have yet to be validated through appropriately designed and preferably phenotype-enriched RCTs.

It is critical for research to focus on identifying the main biological pathways associated with the worsening of kidney function caused by any of several different causes. Furthermore, determining which of the listed pathways can be modified and should therefore be used as a target for treatment is a major area of research interest. The importance of the five mechanisms identified may be more or less pronounced depending on the stage of the disease and patient characteristics.

Despite substantial therapeutic advances, current DKD treatments remain insufficient to fully prevent disease progression, and kidney function decline frequently continues despite guideline-directed, multidrug therapy. While RAAS blockade, SGLT2 inhibitors, GLP-1 RAs, and nsMRAs each confer clinically meaningful benefits, none adequately address the full spectrum of pathogenic mechanisms driving DKD progression.

One of the critical therapeutic gaps is the lack of reliable methods to identify individuals who will be poor responders to standard therapy. Current treatment guidelines predominantly use albuminuria and eGFR to assess the patient’s initial response. These methods do not adequately represent the continued presence of microvascular injury, tubulointerstitial injury, and concomitant fibrosis. There is a need for future studies to focus on validating new biomarkers that will allow for earlier identification of both ongoing structural damage and the likelihood of being a poor responder to treatment.

One fundamental limitation of current therapies is their partial and pathway-specific mechanism of action. RAAS inhibitors and SGLT2 inhibitors primarily target glomerular hemodynamics and metabolic stress, while GLP-1 RAs exert indirect renal benefits largely through systemic metabolic and anti-inflammatory effects, and nsMRAs attenuate mineralocorticoid receptor–mediated inflammation and fibrosis. Although these mechanisms are complementary, they fail to comprehensively suppress key drivers of residual renal risk, including persistent intrarenal inflammation, irreversible fibrotic remodeling, microvascular rarefaction, tubular metabolic dysfunction, and epigenetically programmed injury resulting from prior hyperglycemic exposure [14,51]. As a result, current therapies slow but do not halt the progression of structural kidney damage once these processes are established. Many proposed therapeutic strategies remain supported primarily by preclinical or observational evidence, and prior failures of antifibrotic and immunomodulatory agents caution against assuming that mechanistic relevance will necessarily translate into clinical benefit.

Another major therapeutic gap relates to disease heterogeneity and the timing of intervention. DKD encompasses diverse clinical phenotypes with distinct underlying pathophysiology, yet existing treatment algorithms largely apply a uniform approach based on albuminuria and eGFR. This paradigm inadequately captures non-albuminuric DKD, tubulointerstitial-dominant disease, and rapid progressor phenotypes, which often respond suboptimally to therapies developed primarily for albuminuric, glomerular-centric disease [2,17,50]. Furthermore, many patients initiate renoprotective therapy only after substantial irreversible structural damage has occurred, limiting the capacity of treatment to meaningfully alter long-term outcomes and reinforcing the need for earlier identification of high-risk individuals.

When a patient’s eGFR drops lower than previously thought because they have had the recommended medical interventions performed appropriately according to guidelines, this situation should prompt a formal reassessment of that patient rather than simply continuing with the same treatment options. Clinicians would be especially concerned about further eGFR decline once a patient has received multiple classes of medications, such as ACE inhibitors, angiotensin receptor blockers, SGLT2 inhibitors, GLP-1 RAs, and/or nsMRAs for many months. Clinicians could investigate other reasons for the deterioration of eGFR values, as well as identify other phenotypic characteristics. In addition, clinicians should not experience “therapeutic inertia” when eGFR continues to decline. The recognition that a patient has not responded to a given therapy needs to be combined with realistic goal setting in terms of changed expectations, such as changing from reversing the disease to slowing disease progression and preserving any residual kidney function, particularly if irreversible damage has already occurred.

These observations raise several clinically meaningful hypotheses that warrant prospective evaluation: (i) whether earlier initiation of combination therapy can prevent the establishment of irreversible fibrosis and metabolic memory; (ii) whether phenotype-guided treatment selection improves renal outcomes compared with uniform guideline-based approaches; and (iii) whether treatment goals should shift from albuminuria reduction alone toward stabilization of eGFR slope and preservation of nephron mass.

These limitations underscore the necessity for phenotype-driven and combination therapeutic strategies. Rather than sequential escalation based solely on disease stage, future approaches should aim to match therapies to dominant pathogenic mechanisms within individual patients. Integrating clinical features, biomarkers of inflammation, fibrosis, and tubular injury, and potentially imaging or genetic data could enable more precise risk stratification and guide rational combination therapy [54,71]. While current practice increasingly adopts a layered approach, combining RAAS blockade with SGLT2 inhibitors and nsMRAs, evidence-based frameworks for optimal sequencing, timing, and patient selection remain underdeveloped, representing an important area for future research.

Emerging therapeutic strategies increasingly focus on direct modulation of inflammation and fibrosis, pathways that are central to residual renal risk and only partially addressed by existing agents. Targeted anti-inflammatory approaches, including inhibition of specific cytokines, chemokines, and intracellular signaling pathways such as Janus kinase–signal transducer and activator of transcription pathway (JAK–STAT), have shown promise in early-phase studies, although clinical translation has thus far been limited by safety concerns and modest efficacy [40]. Similarly, antifibrotic strategies aimed at TGF-β signaling, CTGF, or maladaptive repair pathways have yet to yield definitive clinical benefit, highlighting the complexity and redundancy of fibrotic networks in DKD.

More recently, interest has shifted toward therapies that modulate cellular metabolism, mitochondrial function, and maladaptive stress responses, as well as agents that may influence epigenetic programming associated with metabolic memory. These approaches hold theoretical appeal, particularly if introduced early in the disease course, but remain largely investigational [24]. Advances in biomarker development and trial design may facilitate more efficient evaluation of such agents by enabling enrichment of patient populations most likely to benefit and by moving beyond albuminuria-centric endpoints.

Importantly, many of the proposed future therapeutic strategies discussed in this section are grounded in mechanistic rationale and observational associations rather than definitive outcome trial evidence, underscoring the need for appropriately designed RCTs to validate their clinical efficacy.

## 7. Clinical Implications and Conclusions

In conclusion, residual renal risk remains a defining challenge in the management of diabetic nephropathy, even in the era of modern renoprotective therapies. Recognizing the mechanisms and clinical expression of residual renal risk is essential for optimizing patient care and guiding future research and innovation. While current therapies have transformed DKD outcomes, further progress will depend on earlier, more personalized, and mechanism-based approaches that move beyond risk reduction toward durable preservation of kidney structure and function.

These future approaches are supported by strong biological rationale but, in many cases, remain to be confirmed by high-level clinical evidence. Once advanced fibrosis, microvascular rarefaction, and nephron loss are established, currently available therapies can slow, but no longer prevent, the progression of DKD, underscoring a critical point of no return that reinforces the necessity of early identification and timely intervention.

This review adds to the existing international literature by explicitly framing residual renal risk as a distinct clinical and biological state, rather than as incomplete treatment response alone. By contrasting therapeutic success with persistent progression across contemporary trials and clinical phenotypes, this review underscores the need to formally acknowledge residual renal risk in clinical practice, trial design, and guideline development. We propose that recognizing non-albuminuric disease, rapid progressors, and therapeutic non-responders as key contributors to residual renal risk may facilitate more realistic goal setting, improved patient stratification, and the development of targeted, phenotype-driven interventions.

## Figures and Tables

**Table 1:** Contemporary Therapies in Diabetic Kidney Disease and the Persistence of Residual Renal Risk.

Therapeutic Class	Primary Renoprotective Mechanisms	Key Evidence for Renal Benefit	Main Limitations	Contribution to Residual Renal Risk
RAAS blockade (ACE-I/ARBs) [25,26,27,28,29,33,34,35]	Reduction in intraglomerular pressure; antiproteinuric effect; partial anti-fibrotic signaling	Reduced albuminuria and delayed progression to kidney failure in albuminuric DKD	Limited efficacy in non-albuminuric DKD; incomplete suppression of intrarenal RAAS; hyperkalemia and hypotension; no reversal of fibrosis	Progression continues due to persistent inflammation, fibrosis, and tubulointerstitial injury
SGLT2 inhibitors [7,8,42,43,45]	Restoration of tubuloglomerular feedback; reduced hyperfiltration; improved tubular metabolism; reduced hypoxia and inflammation	Consistent reduction in eGFR decline, kidney failure, and renal death across CKD stages	Benefits are additive, not curative; limited reversal of established damage; reduced efficacy in advanced fibrosis; treatment often initiated late	Ongoing progression driven by irreversible structural injury and non-hemodynamic mechanisms
GLP-1 Ras [55,56,57,58,59]	Improved metabolic control; weight loss; blood pressure reduction; anti-inflammatory and endothelial effects	Reduction in albuminuria; modest effects on renal outcomes; FLOW trial shows benefit on kidney endpoints	Limited effect on hard renal outcomes compared with SGLT2 inhibitors; gastrointestinal intolerance; indirect renal mechanisms	Residual risk persists due to the limited impact on fibrosis and tubulointerstitial pathology
nsMRAs (e.g., finerenone) [10,66]	Inhibition of MR-mediated inflammation and fibrosis; reduced oxidative stress	Reduced kidney and cardiovascular outcomes on top of RAAS blockade	Hyperkalemia risk; evidence mainly in albuminuric DKD; no reversal of established fibrosis	Structural damage and microvascular loss remain despite reduced fibrotic signaling
Multidrug combination therapy	Complementary targeting of hemodynamic, metabolic, inflammatory, and fibrotic pathways	Additive risk reduction in contemporary trials and guideline recommendations	Lack of phenotype-driven selection, tolerability issues, and absence of personalized sequencing strategies	Residual risk persists due to disease heterogeneity and late intervention

Abbreviations: RAAS, renin–angiotensin–aldosterone system; ACE-I, angiotensin-converting enzyme inhibitor; ARBs, angiotensin II receptor blockers; DKD, diabetic kidney disease; SGLT2, sodium–glucose cotransporter 2; CKD, chronic kidney disease; GLP-1 Ras, glucagon-like peptide-1 receptor agonists; nsMRA, non-steroidal mineralocorticoid receptor antagonist.

**Table 2:** Mechanisms and Clinical Phenotypes Driving Residual Renal Risk in Diabetic Kidney Disease.

Mechanism/Phenotype	Pathophysiological Features	Clinical Characteristics	Why Are Current Therapies Insufficient?
Persistent inflammation [48,49]	Sustained cytokine activation; macrophage and T-cell infiltration; NF-κB and JAK–STAT signaling	Elevated inflammatory biomarkers; progressive eGFR decline despite treatment	The anti-inflammatory effects of current drugs are indirect and incomplete
Fibrosis and structural irreversibility [27,28,74]	Excess extracellular matrix deposition; myofibroblast activation; nephron loss	Poor response to therapy; continued decline despite albuminuria reduction	Established fibrosis is largely irreversible with current treatments
Tubulointerstitial injury [47,97]	Tubular metabolic stress; mitochondrial dysfunction; tubular atrophy	Non-albuminuric DKD; progression despite controlled albuminuria	Therapies mainly target glomerular pathways
Microvascular rarefaction [97]	Endothelial dysfunction; capillary loss; chronic renal hypoxia	Ischemic DKD phenotype; reduced response to therapy	No current therapy restores microvascular integrity
Metabolic memory [22,40,41,72,80]	Epigenetic reprogramming; persistent oxidative stress	Long diabetes duration; progression despite good glycemic control	Molecular injury persists after glucose normalization
Non-albuminuric DKD	Predominant tubulointerstitial and vascular pathology	Reduced eGFR without albuminuria	Albuminuria-driven treatment paradigms fail to capture risk
Rapid progressors	Accelerated eGFR decline; high inflammatory or fibrotic burden	Early progression to advanced CKD	Conventional monitoring fails to identify early
Late presenters	Advanced CKD at therapy initiation	Limited absolute benefit from therapy	Structural damage is already extensive

Abbreviations: NF-κB, nuclear factor kappa-light-chain-enhancer of activated B cells; JAK–STAT, Janus kinase–signal transducer and activator of transcription pathway; DKD, diabetic kidney disease; eGFR, estimated glomerular filtration rate.

## Data Availability

No new data were created or analyzed in this study.

## Abbreviations

The following abbreviations are used in this manuscript:

ACE-I	Angiotensin-converting enzyme inhibitor
AGEs	Advanced glycation end products
AKI	Acute kidney injury
ARB	Angiotensin II receptor blocker
CKD	Chronic kidney disease
CRP	C-reactive protein
CTGF	Connective tissue growth factor
DKD	Diabetic kidney disease
ECM	Extracellular matrix
eGFR	Estimated glomerular filtration rate
GLP-1 Ras	Glucagon-like peptide-1 receptor agonists
JAK–STAT	Janus kinase–signal transducer and activator of transcription pathway
KIM-1	Kidney injury molecule-1
MCP-1	Monocyte chemoattractant protein-1
MRA	Mineralocorticoid receptor antagonist
NF-κB	Nuclear factor kappa-light-chain-enhancer of activated B cells
NGAL	Neutrophil gelatinase-associated lipocalin
nsMRA	Non-steroidal mineralocorticoid receptor antagonist
RAAS	Renin–angiotensin–aldosterone system
SGK1	Serum/glucocorticoid-regulated kinase 1
SGLT2	Sodium–glucose cotransporter 2
TGF-β	Transforming growth factor beta
TNF-α	Tumor necrosis factor-alpha
Wnt/β-catenin	Wingless-related integration site/beta-catenin signaling pathway
